# Effect of mitochondrial potassium channel on the renal protection mediated by sodium thiosulfate against ethylene glycol induced nephrolithiasis in rat model

**DOI:** 10.1590/S1677-5538.IBJU.2014.0585

**Published:** 2015

**Authors:** N. Baldev, R. Sriram, P.C. Prabu, A. Kurian Gino

**Affiliations:** 1 School of Chemical and Biotechnology, SASTRA University, Thanjavur, Tamil Nadu, India; 2 Vascular Biology Lab, SASTRA University, Thanjavur, Tamil Nadu, India; 3 Central Animal Facility, SASTRA University, Thanjavur, Tamil Nadu, India

**Keywords:** Diazoxide, glibenclamide receptor [Supplementary Concept], Kidney Calculi, Calcium Oxalate, Pathology

## Abstract

**Purpose:**

Sodium thiosulfate (STS) is clinically reported to be a promising drug in preventing nephrolithiasis. However, its mechanism of action remains unclear. In the present study, we investigated the role of mitochondrial KATP channel in the renal protection mediated by STS.

**Materials and Methods:**

Nephrolithiasis was induced in Wistar rats by administrating 0.4% ethylene glycol (EG) along with 1% ammonium chloride for one week in drinking water followed by only 0.75% EG for two weeks. Treatment groups received STS, mitochondrial KATP channel opener and closer exclusively or in combination with STS for two weeks.

**Results:**

Animals treated with STS showed normal renal tissue architecture, supported by near normal serum creatinine, urea and ALP activity. Diazoxide (mitochondria KATP channel opening) treatment to the animal also showed normal renal tissue histology and improved serum chemistry. However, an opposite result was shown by glibenclamide (mitochondria KATP channel closer) treated rats. STS administered along with diazoxide negated the renal protection rendered by diazoxide alone, while it imparted protection to the glibenclamide treated rats, formulating a mitochondria modulated STS action.

**Conclusion:**

The present study confirmed that STS render renal protection not only through chelation and antioxidant effect but also by modulating the mitochondrial KATP channel for preventing urolithiasis.

## INTRODUCTION

Nephrolithiasis is a complex disease of kidney where crystals or foreign bodies can act as nidi, upon which ions from the supersaturated urine form microscopic crystalline structures (1). The formation of these crystals in the tubular fluid, followed by crystal retention and accumulation in the kidney are the pre-requisite for the development of renal stone. Free particle model, fixed particle model and Randall's plaque hypothesis are some well-established hypothesis for stone formation and growth (2). However, molecular understanding of all these theories underlines the importance of oxidative stress in the renal stone formation.

Thiosulfate, an endogenous molecule derived from the metabolism of H_2_S, is reported to have antioxidant and chelation property (3). Sodium thiosulfate, FDA approved drug is used to lessen the side effects of cisplatin (4) and widely used in the emergency treatment of cyanide poisoning (5). Due to the availability of the sulfur group for the donations and free radical scavenging potential, STS can essentially act as an anti-urolithiatic agent (6). Few studies by Asplin and his co-workers and Yatzidis showed that thiosulfate can prevent calcium phosphate nephrolithiasis. However, LaGrange et al., 2009 reported negative results for thiosulfate in the prevention of calcium stone disease (7). In fact, in both studies, the mechanism by which STS affects calcium deposition was not clearly mentioned.

Potassium channels in nephrons have varied functions ranging from maintaining ionic equilibrium to regulating the volume during hypotonic stress environments. Their activation depends on location across the nephrons, as they can be activated either by altering pH, calcium, sodium, chloride or ATP levels. Thus, effects of K^+^ channels are very complex to study (8). The present study was designed to understand the specific role of ATP sensitive potassium channel using diazoxide (channel opener) and glibenclamide (channel closer) in sodium thiosulfate mediated renal protection from ethylene glycol induced nephrolithiasis in a rat model. The calcium chelating potential of STS was evaluated in vitro using gel diffusion method.

## MATERIALS AND METHODS

### Chemicals

Diazoxide was purchased from Sigma-Aldrich. All other commercial reagents used were of analytical grade.

### Animals

All animal experiments were conducted in accordance with the CPCSEA (Committee for the purpose of conduct and supervision of experiments on animals) guidelines, approved by the institutional animal ethical committee (IAEC No. 214/SASTRA/IAEC), Central Animal Facility, SASTRA University. To demonstrate the anti-urolithiasis property and mechanism of action of sodium thiosulfate we used male albino Wistar rats aged 7 to 8 weeks (180-200g). Animals were kept in polycarbonate cages at a controlled temperature of 25±3°C and 60±10% relative humidity with a 12 h each of dark and light cycle. Rats were acclimatized for one week with standard laboratory diet and tap drinking water before the start of experiment.

### Study design

Forty two male Wistar rats were assigned randomly into seven equal groups. All the doses were selected based on previous studies (6, 9).

Group-1 (Normal control): received water ad libitum for 21 days.
Group-2 (Induction control): received 0.4% ethylene glycol (EG) along with 1% ammonium chloride for one week followed by only 0.75% EG in drinking water for two subsequent weeks.
Groups-3 to 7 received the same treatment as group 2 along with the following drug treatments:
Group-3 (STS): received sodium thiosulfate (400mg/Kg b.wt.) intraperitoneally for 21 days.
Group-4 (Diazoxide): received diazoxide (mito K_ATP_ channel opener; 5mg/Kg b.wt.) intraperitoneally for 21 days.
Group-5 (Glibenclamide): received glibenclamide (mito K_ATP_ channel closer; 10mg/ Kg b.wt.) intraperitoneally for 21 days
Group-6 (STS+Diazoxide): received diazoxide 30min. before administration of STS for 21 days.
Group-7 (STS+Glibenclamide): received glibenclamide 30min. before administration of STS for 21 days.

### Biochemical Parameters

Urine samples from all groups were collected using metabolic cages for 24h and analyzed in triplicates for the levels of urea, creatinine and calcium using respective diagnostic kits from Agappe diagnostics Ltd (India). Whole blood was collected from the retro-orbital sinus on the day of necropsy, centrifuged at 10.000×g for 10 min. and serum chemistry analysis was performed in triplicates for calcium, creatinine, urea, ALP using the respective diagnostic kits purchased from Agappe diagnostics Ltd. & Span diagnostics Ltd. (India).

### Antioxidant assays

After necropsy left kidney was cut into four equal sections. Each section was weighed separately, crushed and homogenized in 3mL ice cold Tris buffer (pH=7.4) for performing various assays. Total protein content was measured by Lowry et al., (1956) (10) and used for further calculation. The remaining sample was used for the estimation of various antioxidant levels in kidney homogenate such as TBARS, SOD, GPX and catalase by previously described standard methods (11) while the level of ALP was measured using commercial kit.

### Histopathology

After 21 days of treatment, rats were euthanized by carbon dioxide inhalation followed by cervical dislocation. Immediate laparotomy was performed to collect both the kidneys. Isolated kidneys were cleaned off the extraneous tissue, weighed and rinsed with ice-cold normal saline. A section from both kidneys was fixed with 10% v/v neutral formalin and processed through graded alcohol series and xylene, embedded in paraffin, sectioned at 5μm, and stained with hematoxylin and eosin for histopathological examination under a light microscope. Three kidney tissues per group were analyzed for nephropathy, obstruction and stone deposition.

### In vitro gel diffusion model

To find the inhibitory effect of STS on calcium oxalate stone formation the gel diffusion assay was performed according to Li et al. with minor modifications (12). A microscope slide was uniformly coated with 3mL of 1% agar. After the agar solidified, two pairs of equidistant wells were made perpendicularly. Sodium oxalate and calcium chloride each 20μl was placed in vertical wells. The horizontal wells were filled either with 20μl distilled water as standard or 20μl of STS at varying concentrations. Then the slide was left in a moist chamber for 24h at room temperature. The calcium and oxalate ions diffuse through the gel and form crystals of calcium oxalate, visible as a cloudy streak in the center. The intensity of crystal formation and size of the crystals was dependent on the molarity of the crystal forming solutions employed. Depending on concentration, the inhibitory substances would modify the density and width of the crystal streak. This was carried out in triplicates with different concentrations (200mM, 100mM, 50mM, and 25mM) of STS. The slides were photographed using Gel Documentation System ‘BioRad Chemidoc XRS’. The images were analyzed using Image J software and densitometry plots were obtained. Relative density of the sample with respect to the control was obtained, and the percentage inhibition was calculated by the following formula: inhibition=1-(relative density of the sample/relative density of the control)*100.

## Statistical Analysis

Data was expressed as mean±SD. The comparison between groups, at various time points during the experiment was conducted using ANOVA followed by multiple comparison tests, particularly Dunnett's test using GraphPad Prism software version 5.0.

## RESULTS

Preliminary observations of the rats indicate that ethylene glycol consumption reduced the body weight while the urine output was elevated at the end of 21 days.

### 

#### Urine and Serum chemistry

Table-1 shows the levels of urea, creatinine and calcium in the urine, and their corresponding serum concentration is depicted in Table-2. Induction group rats showed a significant decrease in the concentration of urea, creatinine in urine, while its serum concentration was significantly higher as compared to normal control rats. Administration of rats with STS, diazoxide, glibenclamide+STS exhibits near normal levels of urea, creatinine and calcium in both urine and serum as compared to normal control rats.

**Table 1:** Urine Chemistry.

Parameters	Normal control	Induction control	Treatment groups				
			STS alone	Diazoxide alone	Glibenclamide alone	Diazoxide+STS	Glibenclamide+STS
Urea (mg/mL)	11.12±0.9	6.06 ±1.2*	9.07±1.3	10.77±1.5	7.33±0.8*	8.92±1.3	10.29±1.5
Creatinine (µm/L)	12.13±1.4	6.26±1.5*	9.42±0.4	8.99±2.9	3.94±0.2*	6.93±1.8*	7.24±1.5*
Calcium (mg/24hr)	0.95±0.1	2.44±0.2*	1.10±0.3	0.69±0.1*	1.23±0.3	1.28±0.2	0.60±0.1*

**Group-1** = served as normal control; **Group-2** = as a stone induction control; **Group-3** = was given STS; **Group-4 and Group-5** = were administered diazoxide and glibenclamide respectively and **Groups 6 and 7** = were pretreated with diazoxide and glibenclamide respectively half an hour before administration of STS. Data of all results are presented as mean±SD (*) p<0.05, statistically different from normal controls.

**Table 2:** serum Chemistry.

Parameters	Normal control	Induction control			Treatment groups		
			STS alone	Diazoxide alone	Glibenclamide alone	Diazoxide +STS	Glibenclamide +STS
Urea (mg/dL)	16.50±0.9	41.94±1.1*	18.76±1.2	19.55±1.3	29.93±1.2*	16.42±1.6	13.74±1.3
Creatinine (mg/ dL)	0.35±0.02	1.03±0.07*	0.38±0.02	0.35±0.06	0.58±0.02*	0.38±0.04	0.35±0.06
ALP (U/L)	57.03±2.3	115.96±2.9*	49.25±2.4	50.73±3.2	76.18±2.4*	70.14±4.2*	41.39±2.1
Calcium (mg/dL)	6.21±0.5	5.14±0.9	4.46±0.7*	3.34±0.7*	1.57±0.2*	4.31±0.7*	4.10±0.9*

**Group-1** = served as normal control; **Group-2** = as a stone induction control; **Group-3** = was given STS; **Group-4 and Group-5** = were administered diazoxide and glibenclamide respectively and **Groups 6 and 7** were pretreated with diazoxide and glibenclamide respectively half an hour before administration of STS. Data of all results are presented as mean±SD (*) p<0.05, statistically different from normal controls.

According to Leibovitch, (13) elevated serum ALP is an indicator of kidney dysfunction, and its levels in the blood can be used as an index to assess the effectiveness of the treatment. Administration of STS, diazoxide and glibenclamide+STS to rats reduced the significant elevation of serum ALP activity (Table-2) shown in induction control groups as compared to normal control rats.

#### Antioxidant status

Ethylene glycol administration to the rat was reported to alter the oxidant and antioxidant balance in the kidney and thereby induces nephrolithiasis in two phases. Initially, it causes the production of free radicals and in the later stage, it initiates infiltration of leukocytes (14). Ethylene glycol treatment significantly (P<0.001) increased the TBARS levels, decreased superoxide dismutase and glutathione peroxidase in the induction control group compared to normal rats. The treatment with STS (400mg/kg) to the rats significantly (P<0.05) reduced the TBARS levels and improved the antioxidant enzymes activities compared to group 2 (Table-3).

**Table 3:** Lipid peroxidation and antioxidant levels.

Parameters	Normal control	Induction control	Treatment groups				
			STS alone	Diazoxide alone	Glibenclamide alone	Diazoxide +STS	Glibenclamide +STS
TBARS (mM/100g tissue)	1.78±0.1	4.21±0.5*	2.10±0.4	0.84±0.09*	0.74±0.05*	0.71±0.03*	0.72±0.08*
Superoxide Dismutase (Units/mg protein)	34.7±4.5	17.64±1.2*	31.24±2.4	26.47±1.2*	25.63±1.3*	29.91±2.3	32.30±2.6
Glutathione peroxidase (µg of GSH utilized/min/ mg protein)	22±1.2	12.13±1.1*	19.14±1.6	15.54±1.3*	16.66±1.5*	19.82±1.1	19.57±1.4

**Group-1** = served as normal control; **Group-2** = as a stone induction control; **Group-3** = was given STS; **Group-4 and Group-5** = were administered diazoxide and glibenclamide respectively and **Groups 6 and 7** = were pretreated with diazoxide and glibenclamide respectively half an hour before administration of STS. Data of all results are presented as mean±SD (*) p<0.05, statistically different from normal controls.

#### Histopathology

**Figure 1:**
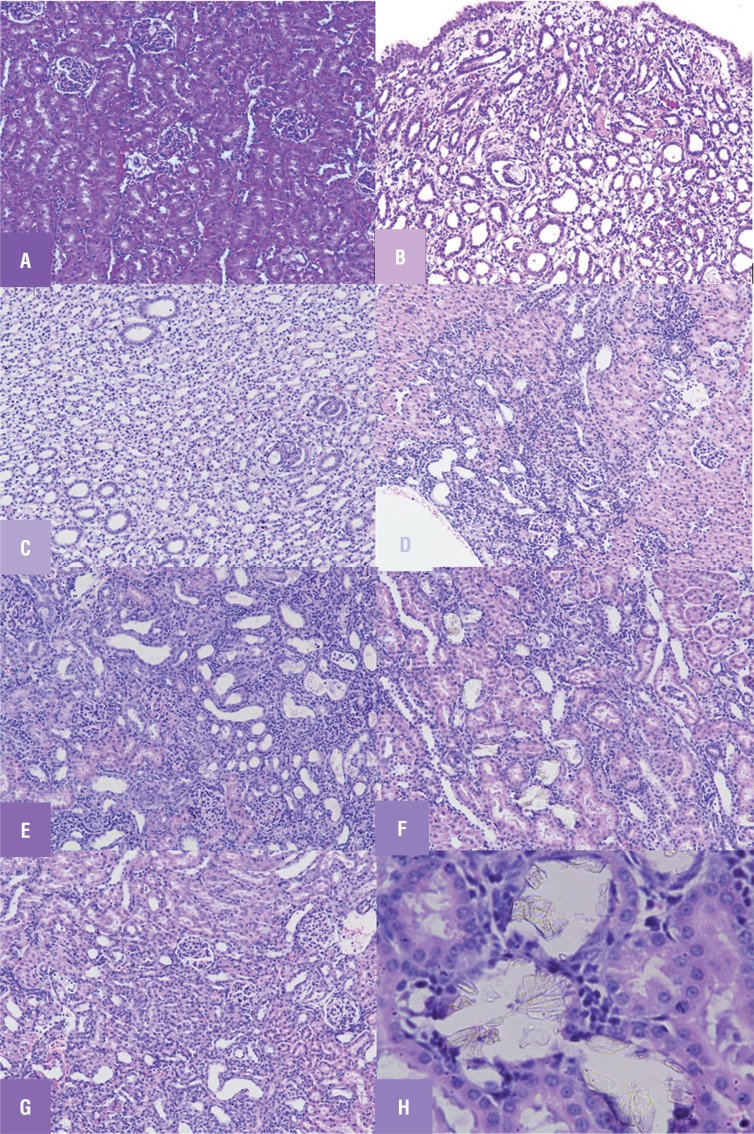
Light microscopic architecture of kidney showing (A) Renal tissue of control (group 1) rats showing no sign of crystal deposition. (B) Renal tissue of urolithiatic rats (group 2) showing crystals deposition and severe obstructive nephropathy (C) Renal tissue of (group 3) STS treated rats showing mild crystal deposition with mild nephropathy. (D) Renal tissue of (group 4) diazoxide treated rats showing mild crystal deposition with moderate obstructive nephropathy (E) Renal tissue of (group 5) glibenclamide treated rats showing prominent crystal deposition with severe obstructive nephropathy (f) Renal tissue of (group 6) diazoxide +STS treated rats showing crystal deposition with severe obstructive nephropathy (g) Renal tissue of (group 7) glibenclamide + STS pretreated rats low crystal deposition with mild nephropathy (h) Crystal's deposition as observed under 40X zoom.

Histopathological analysis of renal tissues in the control group showed no calcium oxalate deposits or other abnormalities in different segments of the nephrons (Figure-1A). But in the urolithiasis induction group, a substantial amount of calcium oxalate deposition was observed, and this was present in whole parts of three major areas of the kidney (Figure-1B). Renal tubular dilations with tubular basophilic and epithelial damage were also observed on pathological examination. In sodium thiosulfate treated group the number of calcium oxalate deposits was significantly lower than that in the disease control group with only mild nephropathy in one of the animals in that group (Figure-1C). Rats treated with diazoxide alone showed less obstructive damage (Figure-1D) while glibenclamide treated rats showed severe damage and obstruction (Figure-1E). Apparently more renal damage, inflammation and hemolysis were observed in rats co-administered with STS and diazoxide (Figure-1F) while STS administration along with glibenclamide showed preserved renal tissue with mild obstruction (Figure-1G).

#### In vitro gel analysis to study chelation effect

**Figure 2:**
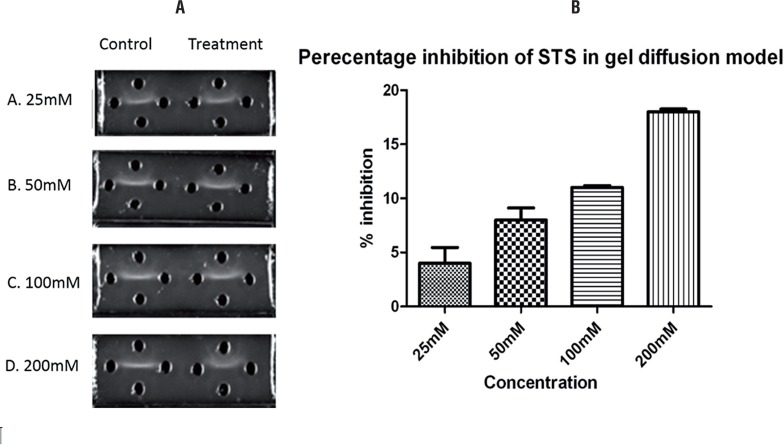
Images of agar gel slides and graph representing the percentage inhibition produced by STS in calcium oxalate crystal formation represented as a streak.

In order to re-confirm the inhibitory effect of STS on calcium oxalate crystal, we performed an in-vitro analysis. The calcium oxalate crystals that have been produced in this study were similar to the crystals in the urine of patients with calcium oxalate crystals. The crystals were predominantly of monohydrate type, confirmed by FTIR. According to Figure-2, STS showed a dose-dependent inhibition of calcium oxalate stone formation. Apparently, only 18% direct inhibition was shown with maximum STS concentration of 200mM, indicating an additional tissue based mechanism for its renal protective action.

## DISCUSSION

Urinary lithiasis is a multifactorial urological disorder that generally occurs as a result of an imbalance between inhibitors and promoters for renal stone formation (15). The human kidney stones predominantly comprised of calcium oxalate, and few studies have examined the effect of the sodium thiosulfate on calcium oxalate crystallization (16) as well. However, the conclusions from those studies were not consistent as few studies claim the beneficial effect of STS and while others, its negative result (6, 7). In the present study, we investigated the effect of sodium thiosulfate on renal stone formation in both in vivo and in vitro models and evaluated its mechanism of action. Our study results were in agreement with previous reports that suggested anti-urolithiatic property of STS but provides a new direction for its mode of action where STS may modulate mitochondrial K_ATP_ channel in rendering renal protection.

Evidence in the literature showed that sodium thiosulfate reduces calcium phosphate stone formation in the genetic hypercalciuric rat (6). However, very little data on the use of sodium thiosulfate for calcium oxalate nephrolithiasis has been published. Adherence of calcium oxalate to renal tubules is associated with free radical mediated injury and the resultant oxidative stress due to hyperoxaluria, which favors crystal adherence (17). Administration of STS to rats for 21 days not only reduced the stress mediated by ethylene glycol, but also prevented the renal dysfunction measured by biochemical parameters like urea and creatinine in urine and serum. Elevated serum alkaline phosphatase activity is considered to be an indicator of renal damage (18). The increased serum ALP activity may be derived from the injury to the brush border membrane of the renal tubular cells (19). The near-normal activity of ALP in the present study was in agreement with the other group that showed a significant decline of ALP activity after STS administration in uremic rats (20). Further evidence for STS protection was confirmed through histological results where papillary crystalline deposits and calcium parenchyma deposits were absent. The direct interaction of STS on calcium oxalate formation was confirmed by using in vitro gel technique and found around 18% inhibition in calcium oxalate formation with 200mM STS, suggesting indirect action of STS in preventing renal stone formation.

A direct co-relation between renal mitochondrial dysfunction and ethylene glycol induced calcium oxalate formation was reported, where the exposure of the proximal tubule with calcium oxalate crystals resulted in rapid and progressive osmotic swelling and dissipation of transmembrane potential of mitochondria resulted in its damage (21). The peroxidation of protein had greater influence on the nucleation and aggregation property of calcium oxalate crystal growth and that predominantly occur in mitochondria (22). Mitochondrial permeability transition pore (mPTP) opening is a terminal event leading to mitochondrial dysfunction and cell death under conditions of oxidative stress. In fact, the vulnerability of the renal tissue towards oxidative stress depends on the functional cross talks between mPTP and mitochondrial K_ATP_ channel (23). In the present study, we evaluated the specific role of mitochondrial potassium ATP channel in the sodium thiosulfate mediated renal protection.

Acccording to previous study results, it is believed that renal protection mediated by sodium thiosulfate is mainly attributed to its chelation and antioxidant potential (6, 24). However, thiosulfates are metabolized in mitochondria, and thus we anticipated a mitochondria based mechanism for its renal protection. In this connection, we used a mitochondrial potassium channel blocker glibenclamide (binds to sulphonylurea receptor subtypes of K_ATP_ channel) and channel opener diazoxide (binds to ATP binding sites of sulphonylurea receptor subtypes of K_ATP_ channel) to evaluate the renal status as supported by biochemical parameters and histopathology (Figure-1, Tables 1–3).

Glibenclamide showed a prominent renal injury as evidenced from altered serum and urine chemistry that was clearly demarcated in histopathology (Figure-1E, Tables 1 and 2). Several lines of evidence showed that glibenclamide can depolarize mitochondrial membrane leading to calcium overload, one of the major factors responsible for free radical release and injury as evident from the lipid peroxidation and antioxidant marker enzyme levels (Table-3). On the other hand, ATP sensitive potassium channel opener, diazoxide treatment showed well-preserved architecture of the kidney (Figure-1D). It prevented mitochondrial swelling and depolarization that may result in permeability pore transition and leads to tissue injury (25, 26). Diazoxide can also modulate the renin angiotensin system, that may play a significant role in developing renal tubule interstitial fibrosis (27) and resulting stone formation as reported with ethylene glycol induced renal injury. Although the mechanism by which the K_ATP_ opener exert their renal protection have not been clarified yet, it is believed that the opening of K_ATP_ channel preserves mitochondrial functional activities through mild uncoupling and depolarization (28). Thus diazoxide mediated protection is an impact on the mitochondria.

In order to confirm the STS mediated mitochondrial K_ATP_ channel modulatory effect, we administered STS along with diazoxide (mitochondrial K_ATP_ channel opener) and glibenclamide (mitochondrial K_ATP_ channel blocker). We found interesting results, where the protective effect shown by diazoxide treatment alone was negated by STS supplementation. On the other hand, STS supplementation to glibenclamide group showed preserved renal tissue architecture. This inverse relationship of STS is an evidence for its interaction with mito K_ATP_ Diazoxide binds to an ATP-sensitive K^+^ transport pathway in kidney mitochondria that affects volume, respiration, and membrane potential and may have a role in the prevention of mitochondrial ATP hydrolysis. Opening of this channel leads to mild uncoupling, blocks calcium entry into mitochondria and leading to renal protection (28, 29). As both diazoxide and STS (mediated through H2S formation) binds to KATP channel in different sites, when diazoxide and STS are given concomitantly, long term or excessive uncoupling may be expected causing ATP hydrolysis and mPTP opening without impairing electron transport, leading to apoptosis. On the other hand, glibenclamide binds to different sulfonylurea subunit blocking potassium entry, thereby exaggerating the ROS production and destabilizing the membrane potential leading to apoptosis (28). When STS is given with glibenclamide, we predict that, H_2_S released from STS may bind to Kir6. 1 subunit of mito K_ATP_ channel, thereby reducing the binding efficiency of glibenclamide resulting its limited action of K_ATP_ channel, allowing STS to mediated its renal protection.

The protective mechanism induced by the opening of mito K_ATP_ is well-studied in cardiovascular diseases. Analogous to the heart system, renal protection by diazoxide may well be claimed due to i) Changes in the mitochondrial Ca^2+^ levels ii) Mitochondrial matrix swelling and changes in ATP synthesis iii) Changes in the ROS levels. Sodium thiosulfate is a known calcium chelating agent with antioxidant properties (30) and can render electrons to complex IV upon its metabolism. Furthermore, several lines of the reports suggest that mitochondrial K_ATP_ channel opening may inhibit mitochondrial permeability transition through inhibiting calcium overload and thereby preserve mitochondrial functions. A proven relationship between mitochondrial membrane potential, mitochondrial dependent apoptosis and calcium overload predicts the possibility of thiosulfate mediated calcium signaling mechanism through calcium/calmodulin-dependent protein kinase for its action, proposed for the future study.

The present study enhances the existing knowledge of STS mediated anti urolithiatic mechanism that emphasizes the calcium chelation and antioxidant property of STS alone. Based on our findings, we suggest that thiosulfate modulate the mitochondrial K_ATP_ channel to render renal protection against stone formation.

## CONCLUSIONS

Based on the results, we found that the administration of sodium thiosulfate effectively prevented the development of urolithiasis in rats, in agreement with the findings of Asplin & Onyeka groups. Even though few mechanisms were proposed earlier for the anti-urolithiasis effect of sodium thiosulfate, no conclusive understanding was reached, and the present study confirm the specific role of ATP sensitive mitochondrial K_ATP_ channel in STS mediated renal protective mechanism.

## Abbreviations

STS =: Sodium thiosulfate
EG =: ethylene Glycol
K_ATP_ =: Potassium ATP channel
mPTP =: mitochondrial permeability transition pore
ROS =: reactive oxygen species
